# Filtering Based Adaptive Visual Odometry Sensor Framework Robust to Blurred Images

**DOI:** 10.3390/s16071040

**Published:** 2016-07-05

**Authors:** Haiying Zhao, Yong Liu, Xiaojia Xie, Yiyi Liao, Xixi Liu

**Affiliations:** 1 Institute of Cyber-Systems and Control, Zhejiang University, Hangzhou 310027, China; zhaohaiying@bupt.edu.cn (H.Z.); zerolover@zju.edu.cn (X.X.); yyliao@iipc.zju.edu.cn (Y.L.); fly02042007@126.com (X.L.); 2 Mobile Media and Cultural Calculation Key Laboratory of Beijing, Century College, Beijing University of Posts and Telecommunications, Beijing 102101, China

**Keywords:** visual odometry, blurred image, adaptive classification, key-frame selection, image gradient distribution

## Abstract

Visual odometry (VO) estimation from blurred image is a challenging problem in practical robot applications, and the blurred images will severely reduce the estimation accuracy of the VO. In this paper, we address the problem of visual odometry estimation from blurred images, and present an adaptive visual odometry estimation framework robust to blurred images. Our approach employs an objective measure of images, named small image gradient distribution (SIGD), to evaluate the blurring degree of the image, then an adaptive blurred image classification algorithm is proposed to recognize the blurred images, finally we propose an anti-blurred key-frame selection algorithm to enable the VO robust to blurred images. We also carried out varied comparable experiments to evaluate the performance of the VO algorithms with our anti-blur framework under varied blurred images, and the experimental results show that our approach can achieve superior performance comparing to the state-of-the-art methods under the condition with blurred images while not increasing too much computation cost to the original VO algorithms.

## 1. Introduction

Visual Odometry [1,2,3,4] employs successive image sequences to estimate the 6 degree of freedom (DOF) poses and can provide a sliding-free odometry measurement for the robots working on uneven roads. Thus the visual odometry can be widely applied to the field rescuer, indoor navigation, space robots, etc. However, most research works on visual odometry are almost based on the assumption that the images sequences obtained from the cameras are clear. In practice, it is hard to guarantee the quality of images as the robots may work on the unknown complex environment. The images will be blurred caused by the violent shaking of camera, and thus decrease the accuracy of the VO significantly. If there are occasional one or two blurred image-frames, VO just can ignore these blurred images. While when the robots are running on the bumped road with insufficient light, images captured in this condition will always exist in varied degrees of blurring. In this condition, if we simply skip all the blurred images, the pose of the camera will lose in the intersection of the road due to feature matching failure.

In order to reduce the harm caused by the blurred images on visual odometry, there are two possible solutions as follows:

Solution 1: Image deblur [5,6,7,8]. Image deblurring methods can be divided into two categories, i.e., blind deblur and non-blind deblur. As to the non-blind deblur, either the blurred kernel of the image or the original information of the image needs to be known. However, the blurred kernels and the original information of the images in visual odometry are usually unknown. Thus the non-blind deblur methods cannot be applied; the blind deblur method tries to estimate the blurred kernel with the point spread function (PSF) iteratively and restore the original image. Those existing blind deblur methods usually need to perform the derivative and convolution operations in the 2D image. And their computational complexity is positive related to the resolution of the images. For example, the method in [5] needs several minutes to deblur an image with a resolution of 640 × 480. In [6], the deblur method is improved with the Fast Fourier Transformation to reduce the temporal complexity, its computation time to deblur an image is still over 40 ms, even the GPU based acceleration is used in their method. In addition, the deblur algorithm will change the pixel information. There is severe ringing effect near the edges of the deblurred image; it may reduce the accuracy of the feature extraction in visual odometry.

Solution 2: Image Quality Evaluation [9,10,11,12]. This solution assesses the quality of the input images, selects the images with better quality feeding to the visual odometry estimation, thereby, it can enhance the accuracy of the odometry estimation. Generally, there are two categories of image quality evaluation methods: subjective evaluation and objective evaluation. The subjective evaluation method mainly relies on the tester’s scores, which stand for the image quality. Obviously, this manual method cannot be applied to the visual odometry. Objective evaluation method evaluates the image quality by a certain objective measure. Comparing with other approaches, this method requires less computation time and can execute without manual operations, thus it may be applied in the visual odometry with blurred images.

In this paper, we address the problem of visual odometry estimation from blurred images, and present an adaptive visual odometry estimation framework robust to blurred images. Our approach employs an objective measure, named small image gradient distribution (SIGD), to evaluate the degree of the blurred image, then an adaptive blurred image classification algorithm is proposed to dynamically recognize the blurred images; finally, an anti-blurred key-frame selection algorithm is proposed to enable the VO robust to blurred images.

The rest of this paper is organized as follows: the related visual odometry solutions for blurred images are given in Section 2; our robust visual odometry approach is presented in Section 3; in Section 4, simulation and real data experiments are carried out to evaluate the performance of our approach comparing with state-of-the-art methods; finally, the conclusion and a further discussion are given in Section 5.

## 2. Related Work

There are few works focusing on the visual odometry anti-blurred images. As image deblurring based methods are time-consuming, researchers introduce data association based method to accelerate the image deblurring and reduce the blurred images’ effect on visual odometry. For example, the method in [13] uses the simultaneous localization and mapping (SLAM) to rebuild the three dimensional structure and calculate the motion of the camera, then the initial blurred model parameters of the blurred image are obtained by the previous structure and motion. Finally, the image can be restored by the fast deconvolution method based on those initial blurred model parameters. Obviously, the main drawback of this kind of method is temporal complexity, as the SLAM and the deconvolution are time-consuming.

The most related work is the robust visual odometry framework for blurred image presented by Alberto Pretto et al. [14]. In that framework, the kernels’ parameters of the blurred images caused by the motions are estimated firstly, then those parameters are combined into the SIFT based feature detection to improve the accuracy of feature points. To reduce the damage on the feature points’ accuracy caused by the motion blurring, Alberto Pretto’s method keeps the parameters of the Gaussian filter orthogonalized with the direction of the motion unchanged, and the parameters of the Gaussian filter paralleled with the direction of the blur motion are solved with iterative method. This method can reduce the effects of the motion blurred images on visual odometry in a certain degree, but it is limited to the rectilinear motion blurred images and Gaussian filter based feature detection methods. There are other kinds of image blurring, such as the focusing blur, nonlinear motion blur, etc., all of these are beyond the scope of their framework. In addition, there are also many different feature detection methods [4], such as Harris corner detection, FAST corner detection, all of these detectors cannot be used in their framework. Furthermore, their method employs the Levenberg-Marquardt Algorithm (LMA) to search a suitable Gaussian standard deviation, the optimization results of LMA are great affected by the initial values, thus it may lead to varied results when the input initial values are slightly changed.

There are also some other approaches, which try to optimize the motions of the robots to avoid the condition of image blur. For example, the work in [15] continuously adjusted the navigation actions of the robot, and used the reinforcement learning method to choose better navigation actions for the robot to avoid image blur. The work in [16] tried to reduce the motion blur by installing the camera along the most stable visual direction. These kinds of approaches do not process the blur images in the visual odometry directly, thus it may be constrained in many conditions.

## 3. Visual Odometry Robust to Blurred Images

The blurred images will decrease the accuracies of the extracted features significantly, thus lead to large bias on the estimation of the visual odometry. As the computational complexity of the visual odometry is large, the image quality evaluation algorithm must have highly computation efficiency in order to guarantee the real-time performance of the visual odometry with blurred images. In the visual odometry, the image sequences are similar, thus image quality evaluation algorithm should also be able to distinguish the blurred images from those similar images.

**Figure 1:**
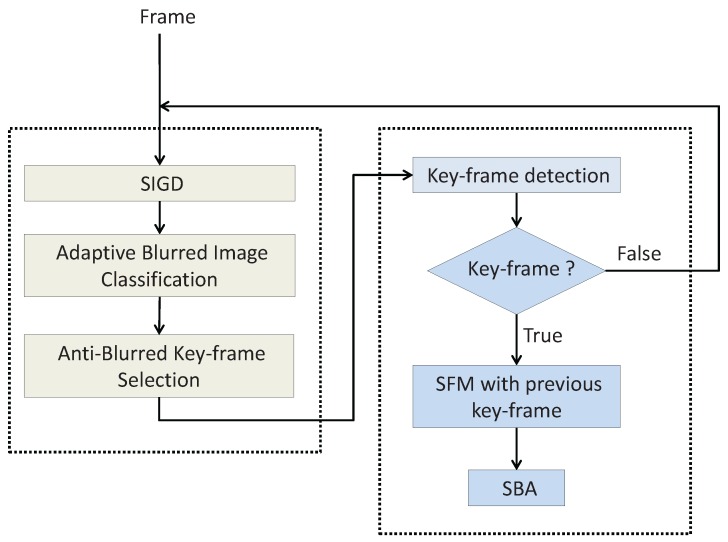
Framework of our robust visual odometry anti-blurred image. The left box is our framework, and the right box is a typical pipeline for the VO, and the output of our framework can be easily regarded as the input of the VO algorithms. Here SFM is the abbreviation of Structure From Motion [17], and SBA is the abbreviation of Sparse Bundle Adjustment.

Our robust visual odometry anti-blurred image framework is shown in Figure 1. Firstly, we present a SIGD algorithm (Algorithm 1) to calculate the blurred degree of each image frame. Then an adaptive blurred image classification algorithm (Algorithm 2) is applied to the image frames based on the blurred degree of the image frame and the blurred degrees of the successive image frames. The adaptive blurred image classification algorithm will divide all the image frames into two categories, i.e., clear set C1 and blurred set C2. Finally, an anti-blurred key-frame selection algorithm (Algorithm 3) is proposed to improve the VO’s capability for anti-blurred images based on the divided categories.

### 3.1. Blurred Degree Calculation with SIGD

One of the reasons that the blurred image occurs is that there is a relative quick motion between the target object and the camera during the exposure. Then the blurred image can be formally denoted as:

(1) g(u,v)=h(u,v)*f(u,v)+n(u,v)

where h(u,v) is the point spread function (PSF), n(u,v) is a random noise. With a convolution of the blurred kernel, the image will lose a lot of details in the high frequency, and the image edges become flat. Normally, the gradient magnitude of the sharp edge is larger than that of the flat edge. In this paper, we present a real-time small image gradient distribution (SIGD) based blurred degree evaluation algorithm, which quantitatively concerns the differences between the blurred images and the clear images.

**Figure 2:**
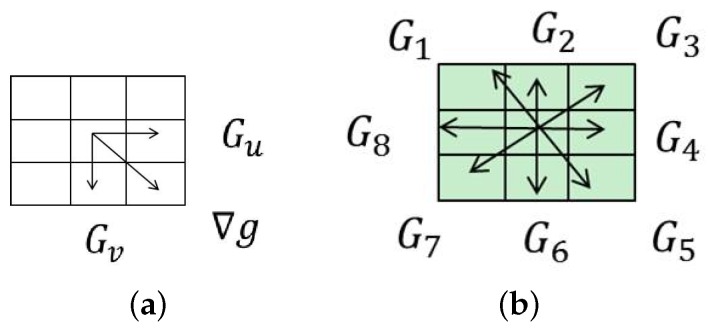
The original and our improved image gradient calculation methods. (**a**) Original gradient Calculation; (**b**) Improved gradient Calculation.

The traditional image gradient calculation methods, such as Figure 2a, which only concern the pixel-wise differences in two directions, cannot deal with the rotation motion blurring. In order to reduce the efforts of the relative rotation between cameras and objects, we present an improved image gradient calculation method, as shown in Figure 2b. In our method, we calculate the differences (denoted as $G_{i},i=1,2,...,8$) along the eight directions for the center point of $\left(u_{0},v_{0}\right)$ within the range of 3 × 3 pixels, and we take the maximum absolute value of those differences in eight directions as the gradient on $\left(u_{0},v_{0}\right)$. In order to reduce the interference of colors, we only use the Y channel in YUV color space that emphasizes the brightness channel, and the gradient in Y channel is denoted as $\Delta g_{Y}\left(u,v\right)$, calculated with Formula (2).

(2) $$\begin{array}{c} \Delta g_{Y}\left(u,v\right)=max(|G_{i}\left|\right)\;\left(i=1,2,\cdots ,8\right)\\ =max(|Y(u,v)-Y(u-1,v-1)|,|Y(u,v)-Y(u,v-1)|,\\ |Y(u,v)-Y(u+1,v-1)|,|Y(u,v)-Y(u+1,v)|,\\ |Y(u,v)-Y(u+1,v+1)|,|Y(u,v)-Y(u,v+1)|,\\ \left|Y(u,v)-Y(u-1,v+1)|,|Y(u,v)-Y(u-1,v)|\right) \end{array}$$

The blurred degree of each image frame, denoted as *b*, is calculated with Formulas (3) and (4).

(3) $$b=\frac{10}{M*N}\sum_{u=1}^{M}\sum_{v=1}^{N}l(u,v)$$

(4) $$l\left(u,v\right)=\left\{\begin{array}{c} 1,\text{if}\;\Delta g_{Y}\left(u,v\right)⩽B\\ 0,\text{if}\;\Delta g_{Y}\left(u,v\right)>B \end{array}\right.$$

where *M* and *N* are the width and height of the image, $\Delta g_{Y}\left(u,v\right)<B$ is the rotation invariant gradient at the point of (u,v). *B* is a gradient magnitude threshold.

The blurred degree of the image frame can be regarded as the number of the pixels whose gradients are not larger than the threshold *B*. Considering that the number of pixels is related with the resolution of the image, we normalize it into a range of [0, 10]. The larger *b* represents higher blurred degree on the image. *B* is an experimental threshold and normally set as [8,10] in our approach.

Then the SIGD based blurred degree calculation algorithm can be summarized as Algorithm 1.

**Algorithm 1:** Blurred Degree Calculation with SIGD **Input**: Image g(u,v), Gradient threshold *B* **Output**: Blurred degree *b* 1 Detect *M* and *N* of g(u,v) 2 Convert g(u,v) to YUV color space, construct $g_{Y}\left(u,v\right)$ 3 For each pixel in $g_{Y}\left(u,v\right)$, calculate its $\Delta g_{Y}\left(u,v\right)$
4 Calculate blurred degree *b* of $g_{Y}\left(u,v\right)$ by Formulas (3) and (4)

### 3.2. Adaptive Blurred Image Classification

In the visual odometry, we cannot use a constant blurred degree threshold, denoted as $\hat{b}$, to classify the images (With constant threshold, the image will be labeled to a blurred image, once the image’s blurred degree is larger than the constant threshold $\hat{b}$.), as the surrounding environment is constantly changing. This means the different scenes may cause varied blurred degrees even though all the images are not blurred. Fortunately, the changes of the scenes are continuous, then the blurred degrees will not change significantly except that there are sudden blurred images.

As the blurred degree of the blurred image will be greater than that of the clear image, the low-pass filter, such as the inertial filter, can be considered to filter the blurred images. However, there are serious phase lags on the inertial filter, which leads to that the inertial filter cannot follow the changes of the environment and misclassifies the blurred images. Although the finite impulse response (FIR) filter has linear phase, its output can only reflect the input of the system, while it cannot reflect the previous output of the system.

In this paper, we combine the advantages of both the inertial filter and the FIR filter to propose an adaptive blurred image classification filter as follows.

(5) $$y_{n}=\gamma y_{n-1}+\left(1-\gamma \right)\left(\frac{1}{S}\sum_{s=1}^{S}x_{n-s}+\beta \right)$$

where $y_{n}$ is the current output of the filter, here it refers to the current classification threshold $K_{n}$. $y_{n-1}$ is the previous output of the filter, here it refers to the previous classification thresholds $K_{n-1}$. $x_{n-S},\cdots ,x_{n-1}$ are the input values of the system, here they refer to the blurred degrees of the images, $b_{n-S},\cdots ,b_{n-1}$.

By changing the values of *γ*, we can adjust the ratio between the inertial filter and the FIR filter. *S* is the window size of the sampling, *β* is the bias fact.

Then the adaptive blurred image classification algorithm can be summarized as Algorithm 2.

**Algorithm 2:** Adaptive Blurred Image Classification **Input**: Image sequence $g_{1},g_{2},\cdots ,g_{n}$ 1  **Initialization:** scalar factor *γ*, window size *S*, bias parameters *β*, $K_{0}=0$ 2  **for**
i=1,⋯,n
**do** 3  **if**
i>S
**then** 4    $K_{i}=\gamma K_{i-1}+\left(1-\gamma \right)\left(\frac{1}{S}\sum_{s=1}^{S}b_{i-s}+\beta \right)$ 5  **else** 6    **if**
i==S
**then** 7      $K_{i}=K_{i}/S$ 8    **else** 9      $K_{i}=K_{i-1}+b_{i}$ 10    **end** 11  **end** 12  **if**
$b_{i}>K_{i}$
**then** 13    $g_{i}$ is blurred 14  **else** 15    $g_{i}$ is clear 16  **end** 17 **end**

### 3.3. Anti-Blurred Key-Frame Selection for Robust Visual Odometry

As mentioned in previous sections, our anti-blurred approach is based on a key-frame selection police and try to reduce the damages caused by the blurred images with selected key-frames feeding to the motion estimation of the VO. This section presents an anti-blurred key-frame selection algorithm for the robust visual odometry.

Most of the blurred images in the VO applications are caused by the robots walking fast in the rough road under complex light situations. There is only a relative small motion between two successive image frames, which will accumulate errors in the motion estimation, we then use key-frame based estimation to reduce the harm caused by both the relative small motion and the blurred images.

As there may be varied key-frame selection polices [18,19], our anti-blurred key-frame selection algorithm is designed to support varied key-frame selection policies. In our anti-blurred key-frame selection algorithm, we provide a basic key-frame selection principle, which is based on the motion of two frames. Then given the current key-frame, the next key-frame in the frame sequence can be roughly calculated with the following formula based on the principle of the relative motion.

(6) $$D_{min}⩽\left|T_{N_{next}}-T_{N_{current}}\right|⩽D_{max}$$

Here $T_{N_{next}}$ and $T_{N_{current}}$ are the translations of the frame $N_{next}$ and the frame $N_{current}$ respect to the global coordinate. $D_{min}$ and $D_{max}$ are the thresholds for the margin of the motion, and can be experimental set based on the velocity of the robots in practice.

As the Formula (6) is only a rough estimation for the next key-frame, we only use it to constrain the range of the candidate key-frames, which means the image frames, whose relative motions to the previous key-frame located in the distance interval of Formula (6), are all possible selected as the next key-frame.

We then introduce the key-frame selection algorithm to anti-blurred images. Based on the Algorithm 2, we can adaptive classified the image frames into two categories, denoted as C1 and C2. C1 set contains all the images that have smaller blurred degrees than their corresponding blurred image thresholds, and the remainder images are divided in to C2. Then C1 set refers to the clear images and C2 set refers to the blurred images. Thus the anti-blurred key-frame selection algorithm is presented as follow.

**Algorithm 3:** Anti-blurred Key-frame Selection **Input**: Image sequence $g_{1},g_{2},\cdots ,g_{n}$, $N_{current}$, *D* 1  **for**
*each*
$i>N_{current}\;\&\&\;D_{min}⩽\left|T_{g_{i}}-T_{N_{current}}\right|⩽D_{max}$
**do** 2  Calculate blurred degree $b_{i}$ and its threshold ${\hat{b}}_{i}$ 3  **if**
$b_{i}⩽{\hat{b}}_{i}$
**then**
4    Push($g_{i}$,C1) 5  **else**
6    Push($g_{i}$,C2) 7  **end** 8  **end** 9  **if**
*C1 != NULL*
**then** 10  return Pop(C1) 11 **else** 12  Sort(C2) 13  return Pop(C2) 14 **end**

In Algorithm 3, the step 1 is used to evaluate whether the current frame is a candidate of the next key-frame, then we can apply other key-frame selection policies, such as the policies in [19], to our Algorithm 3 via updating the evaluation metrics in step 1. In our Algorithm 3, the frame $g_{i}$’s blurred degree is denoted as $b_{i}$, which can be calculate by Algorithm 1; the frame $g_{i}$’s blurred degree threshold is denoted as ${\hat{b}}_{i}$, which can be calculate by Algorithm 2. Push($g_{i}$, C1) is the operation to push the image $g_{i}$ into the stack of C1, Pop(C1) is the operation to pop the top element from the stack of C1, and Sort(C2) is the operation to sort all the elements in C2 with an ascending order. Thus the operation of Pop(C2) in step 13 will pop the element with the smallest blurred degree.

We carry out different selection principles for these two sets C1 and C2 in Algorithm 3. If there are elements in C1, we will return the frame that is less than its corresponding blurred degree threshold and most far away from $N_{current}$. If the C1 is empty, we will return the clearest frame in C2, it is also the frame with the smallest blurred degree in C2.

## 4. Experiments

In order to validate the adaptive blurred image classification algorithm and the anti-blurred key-frame selection algorithm, we carry out experiments in both the open benchmark dataset and the real datasets captured by our mobile platform. The real datasets are captured by our small mobile platform equipped with a stereo camera system.

### 4.1. Performance Evaluation for SIGD

This experiment is to evaluate the discrimination of our SIGD [20], we compare our SIGD with several state-of-the-art methods [21], such as Marziliano [10], JNBM [11], and CPBD [12], which are classical metrics for blurred image detection.

As suggested in [22], the blurred degree calculated by a good measuring algorithm should be monotone on the blurred degrees for real blurred images. To evaluate different blurred degree metrics, we generate a series of blurred images from a same original clear image, these blurred images are ranked with the increasing of their real blurred degrees. In this section, three blurred types, i.e., motion blur, rotation blur and Gaussian blur, are considered as they are commonly occurred in the out-door environment.

#### 4.1.1. Linear Motion Blurred Image

**Figure 3:**
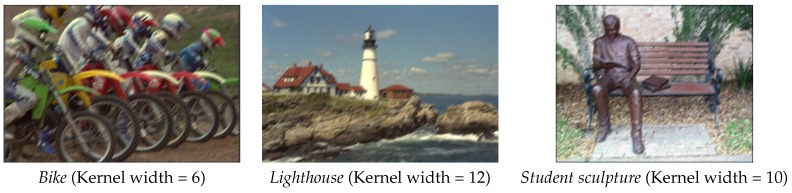
Three linear motion blurred images used in our experiments.

Three examples of the linear motion blurred images are given in Figure 3. The linear motion blur kernel can be depicted by its direction and width. In this experiments, a series of linear motion blurred images are generated with increasing kernel width and fixed direction as shown in Table 1. The estimated blurred degree of our SIGD are given in Table 1, comparing with Marziliano, JNBM, and CPBD. Results show that all these four metrics are monotone to the linear motion degree (direct proportion for SIGD and Marziliano and inverse proportion for JNBM and CPBD), which means they are able to reflect the real linear motion degree.

#### 4.1.2. Gaussian Blurred Image

**Figure 4:**
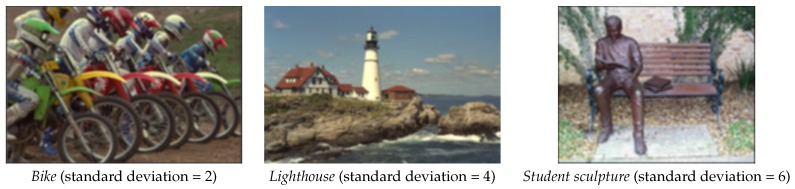
Three gaussian motion blurred images used in our experiments.

Figure 4 gives three examples of Gaussian blurred images, whose blurred degrees mainly depend on the standard deviation of Gaussian kernel. In Table 2, we evaluate the performances of the four metrics on the Gaussian blurred images with increasing standard deviation. As can be seen, SIGD is directly monotone to the real blurred degree, JNBM is inversely monotone to the degree of the blurred images. However, the estimated blurred degrees output by Marziliano and CPBD have some inconsistent fluctuations with the growing of real blurred degree.

#### 4.1.3. Rotation Motion Blurred Image

**Figure 5:**
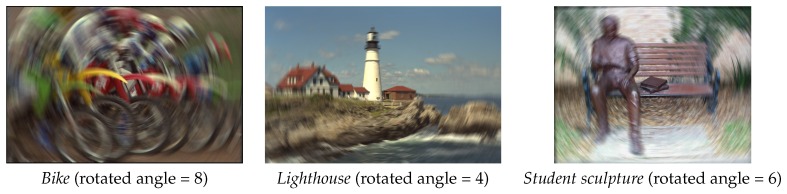
Three rotation motion blurred images used in our experiments.

Rotation motion blur is another common type in image blurring, which is illustrated in Figure 5. Here the rotation angle is increased to generate the test images with known rotation blurred degrees. Table 3 depicts the performances of four metrics on the rotation motion blurred images. In this experiment, SIGD is still directly monotone to the real blurred degree. As for the other three metrics, they go ups and downs as the rotation angle keeps increasing.

#### 4.1.4. Computational Time Evaluation

**Figure 6:**
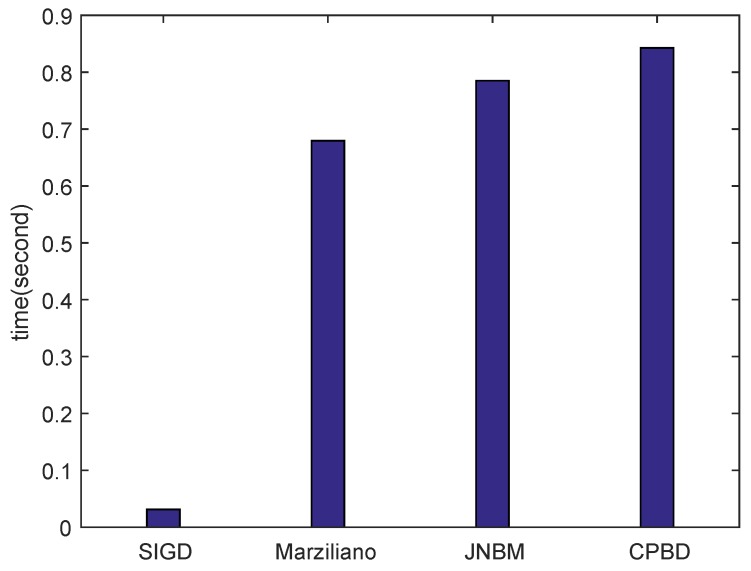
Average computation time for each method.

We also compare the mean computational time of different algorithms since high efficiency is important when evaluating the blurred degree. As shown in Figure 6, SIGD is significantly efficient compared to other three algorithms.

The overall above experimental results show SIGD can give reliable consistent evaluation to the blurred images with respect to linear motion blur, Gaussian blur and rotation motion blur. Furthermore, there is quite less computational cost required by SIGD compared to other state-of-the-art metrics, which is especially suitable for the VO estimation in real-time.

### 4.2. Evaluation for Adaptive Blurred Image Classification

In this section, we employ both the open benchmark dataset and the real dataset to evaluate that our adaptive blurred image classification algorithm (Algorithm 2) [20] can filter the relative clear images into the C1 and reject the blurred images, and our Algorithm 2 is also robust to the changes of the scenes in varied environments. In the following experiments of this paper, our SIGD uses the same parameter settings. The window size S is set to 5, the *γ* in Formula (5) is set to 0.94 and the bias fact *β*, which is a constant inversely proportion to the resolution of the image, is set to 100,000/(M× N). Here M and N is the width and height of the input image.

#### 4.2.1. Experiments on Benchmark Datasets

**Figure 7:**
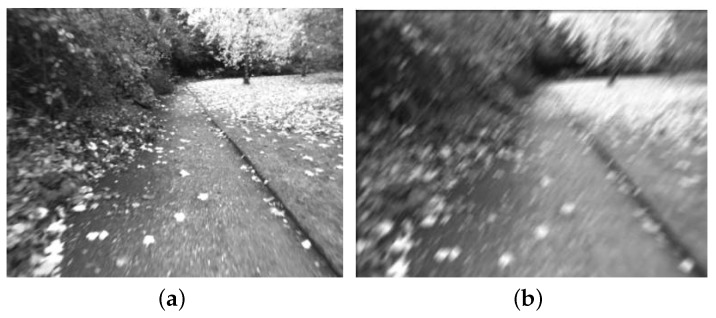
(**a**) Clear image sample in the original NewCollege dataset; (**b**) Image sample after adding blurred noise in the NewCollege dataset.

**Figure 8:**
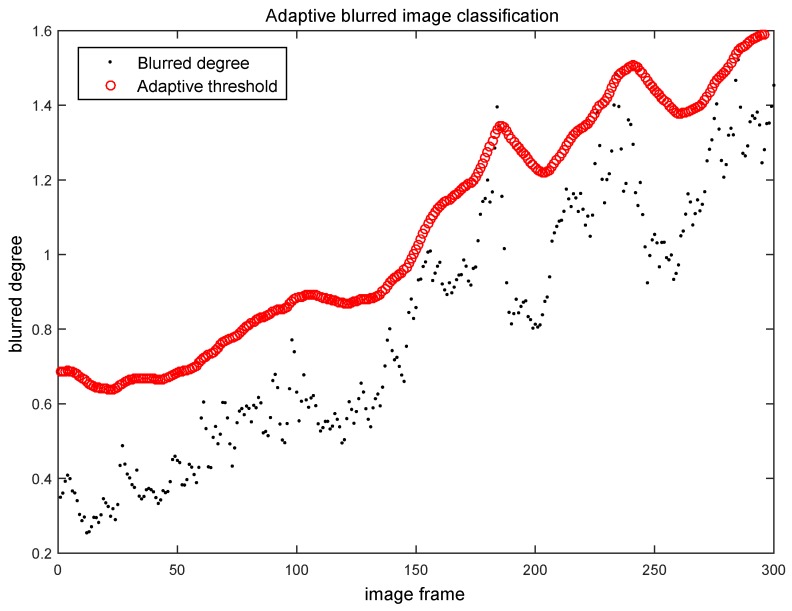
Blurred image classification results on the original NewCollege dataset.

The first evaluation experiment is carried on the NewCollege dataset [23], which is captured by the stereo camera system with a resolution of 512 × 384 on the outdoor road of the Oxford University. Most of the continuous images in that dataset are clear, only few image frames are blurred. A clear image example is shown in Figure 7a. In our experiments, we extract 300 continuous image frames from the NewCollege dataset and use them to evaluate the adaptive blurred image classification algorithm. The classification results of our Algorithm 2 on these 300 image frames are shown in Figure 8. Each black point in Figure 8 represents the blurred degree of the current frame calculated by Algorithm 1. Each red circle in Figure 8 represents the adaptive threshold for the current frame output by Algorithm 2. The results in Figure 8 show that most of the frames’ blurred degrees locate below their corresponding adaptive thresholds, which indicates that most of the images will be classified to be clear, which is consistent with the actual situation in the NewCollege dataset.

In order to further evaluate the blurred image classification algorithm’s performance on the severe blurred image sequence, we add some random motion blurred noises to the original NewCollege dataset. We select 200 successive image frames from 51 to 250. The noise for each selected frame is generated by a set of random parameters, i.e., the blurred width and the blurred direction, which satisfy the Gaussian distributions. A sample of the noised image is shown Figure 7b.

**Figure 9:**
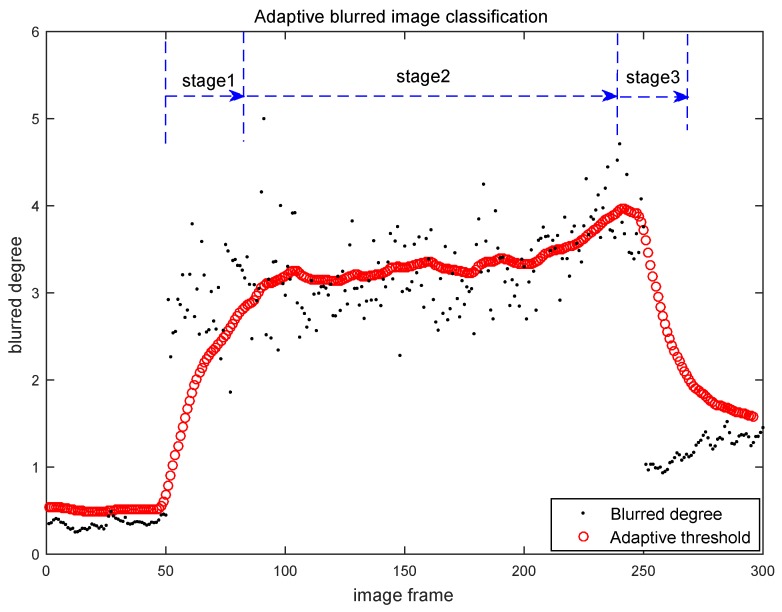
Blurred image classification results on the noised NewCollege data set.

The classification results on the noised dataset are shown in Figure 9. Each black point in Figure 9 represents the blurred degree of the current frame calculated by Algorithm 1. Each red circle in Figure 9 represents the adaptive threshold for the current frame output by Algorithm 2. In the stage 1 of the Figure 9, although the blurred degrees of the images have a sudden increment, our Algorithm 2 can still quickly follow the increment and adaptive update the thresholds. In stage 3 of the Figure 9, our Algorithm 2 can also follow the sudden decreasing and classify the image frames correctly. This jump of the blurred degrees on the image sequence is quite typical in practical visual odometry cases, as the robots may walk through a sudden rough road. In this condition, if all those frames are classified as blurred images and ignored in the selection of key-frames, the motion estimation in VO may fail due to lack of matched features in a large interval of the frames. Based on the same consideration, our algorithm 2 will not regard all the frames in stage 2 as blurred images of C2.

#### 4.2.2. Experiments on Real Captured Datasets

**Figure 10:**
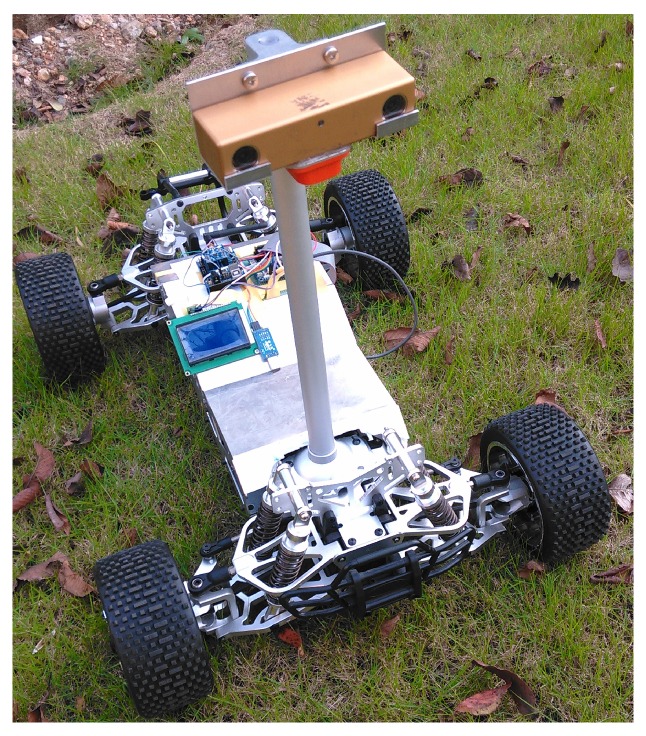
Mobile robot platform used in our experiments, the images are captured by the bumblebee stereo camera system.

**Figure 11:**
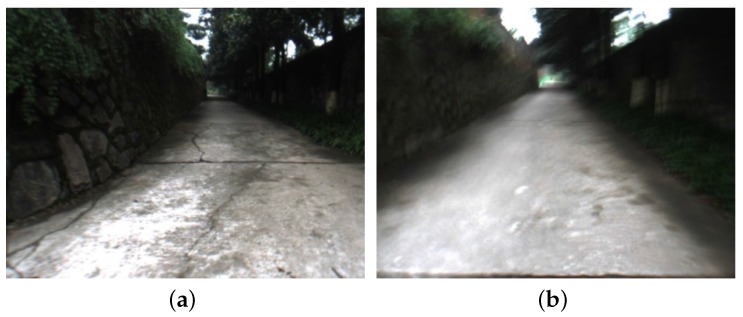
Image samples captured by our platform. (**a**) clear image; (**b**) blurred image.

In this section, we also carry out the evaluation experiment for Algorithm 2 in real captured dataset. We use a small platform, shown in Figure 10, to walk in our campus and capture a set of image frames. In the ahead of these image frames, the platform walks downhill, in the later of these image frames, the platform walks uphill. As the road is quite rough, there are many blurred images in this image sequence, some samples are shown in Figure 11.

**Figure 12:**
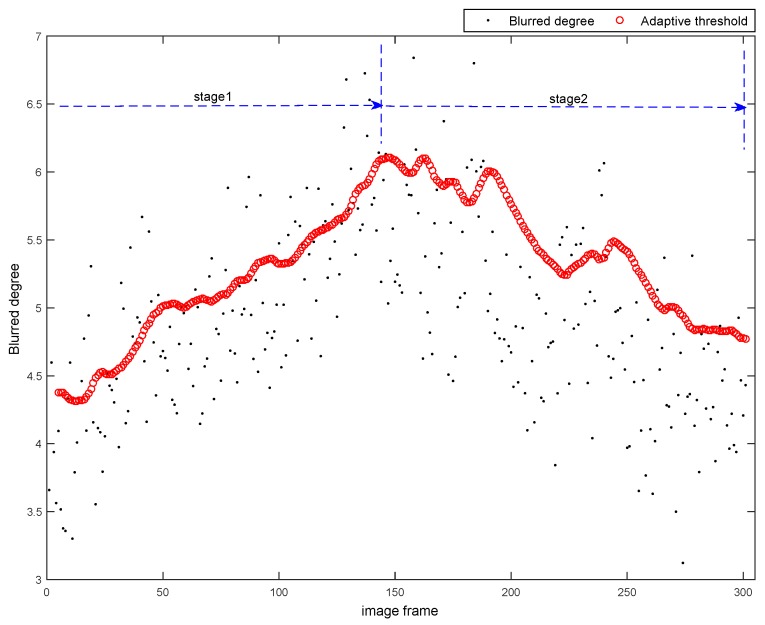
Classification results on the dataset captured by our platform.

The classification results on the real captured data set are shown in Figure 12. Each black point in Figure 12 represents the blurred degree of the current frame calculated by Algorithm 1. Each red circle in Figure 12 represents the adaptive threshold for the current frame output by Algorithm 2.

The Figure 12 shows that the blurred degrees are increasing in the stage 1 and decreasing in the stage 2, which are corresponding to the platform’s downhill (acceleration) and uphill (deceleration) motions. During the stage 1, the thresholds calculated by Algorithm 2 are increased with the growth of the blurred degrees due to the acceleration of the platform. Thus our Algorithm 2 can keep the relative clear images into the C1 and reject the relative blurred images. Similarly, in the stage 2, the thresholds calculated by Algorithm 2 are decreased with the drop of the blurred degrees due to the deceleration of the platform. Our algorithm can also keep the relative clear images into the C1 and reject the relative blurred images.

### 4.3. Evaluation for Visual Odometry with Blurred Images

**Figure 13:**
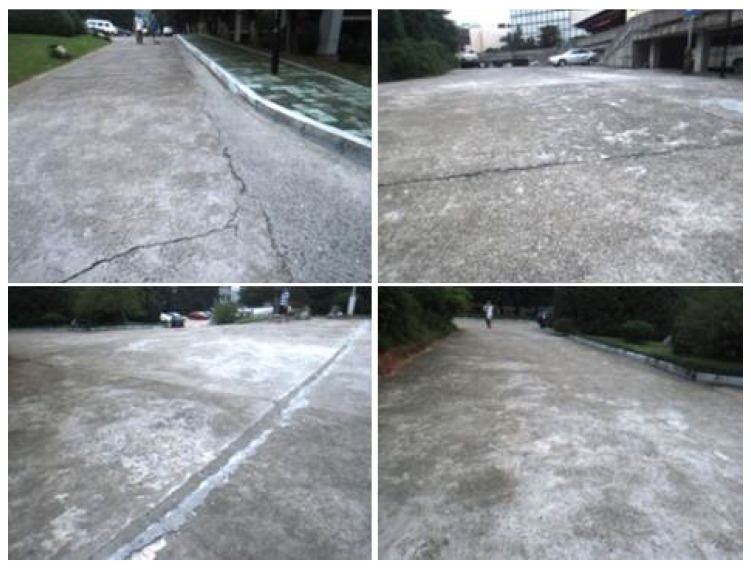
Image samples on dataset1.

**Figure 14:**
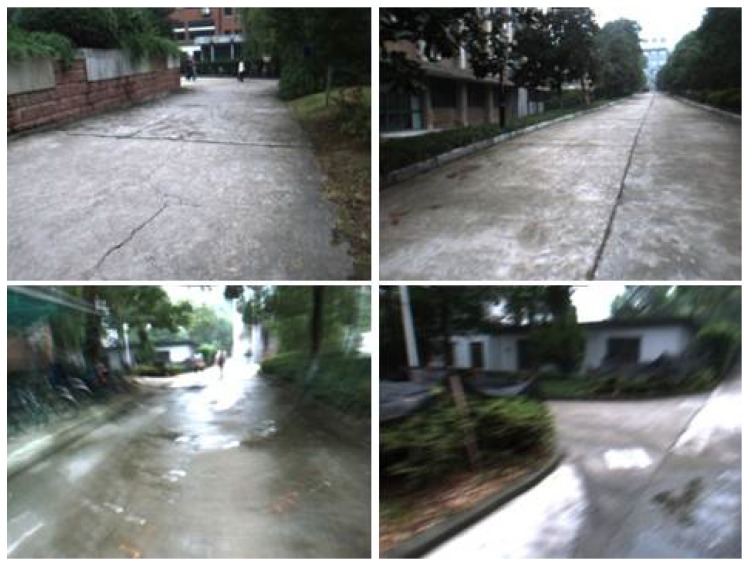
Image samples on dataset2.

In this section, comparable VO estimation experiments on real image datasets are carried out to evaluate the performance of our approach. We use two closed-loop image datasets, dataset1 and dataset2 (These two datasets and our code for algorithm 3 can be downloaded from our website [20]), which are captured by our mobile platform walking a closed circle and stopping at the start point on our campus. There are many blurred images in these two datasets, as the road is rough and the light conditions are changed frequently on the road. Some image samples of these two datasets are shown in Figure 13 and Figure 14.

We also employ two basic visual odometry algorithms in our experiments. The first one is the opensource libviso2 [24] and the second one is a Stereo Visual Odometry (SVO) algorithm [25]. The SVO uses CenSurE detector [26] to detect the interesting points. A SSD(Sum of Squared Difference) based stereo match is then applied on those interesting points and the 3D structures of those matched point-pairs are also calculated by the triangle principle. We then use the SURF descriptor [27] and the RANSAC method to find all the matched points between two adjacent frames. Finally, 2D-3D estimation is applied to obtain the 6D motion.

As these two basic VO algorithms do not concern the frame selection policy, there may be three executing modes as follows.

(a) All the image frames are fed to the VO algorithms. It means the motion is calculated with frame-by-frame based estimation, which is denoted as ***F-B-F*** (frame-by-frame) in the following experiments.
(b) Only the key-frames are fed to the VO algorithms. It means the motion is calculated with key-frame based estimation, which is denoted as ***K-F*** (key-frame) in the following experiments.
(c) The key-frames fed to the VO algorithms are selected by the algorithm 3 presented in this paper. It means the motion is calculated with anti-blurred key-frame selection based estimation, which is denoted as ***A-B*** (anti-blurred) in the following experiments.

In our first evaluation experiment for visual odometry with blurred images, we use the closed-loop error to evaluate these two VO algorithms executing with the above three modes on dataset1 and dataset2. The experimental results are shown in Table 4 and Table 5.

**Figure 15:**
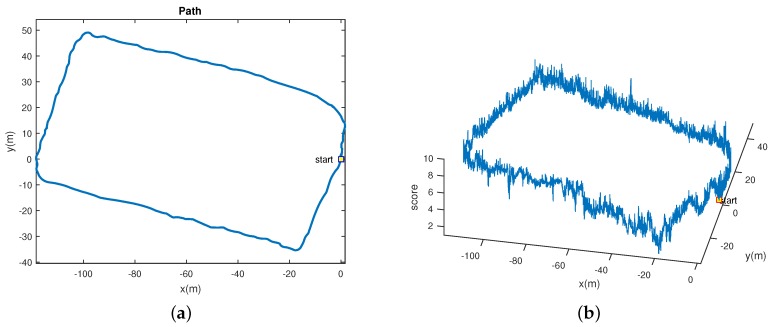
The visualization of the path for the robot walking on dataset2. The third coordinate (`score’) displays the SIGD value of each frame in the path. (**a**) Path in two dimension; (**b**) Path with SIGD.

The experimental results in both tables show that our anti-blurred approach can achieve the best performance in both datasets with both VO algorithms. And we also find that both the VO algorithms executing with the key-frame mode can achieve better performances than that with the frame-by-frame mode. The experimental results also show that our anti-blurred approach can significantly promote the performances of both VO algorithms in dataset2, while it only slightly promote the performances of these two VO algorithms in dataset1. After a further analysis, we find that there are less blurred images in dataset1, which may indicate that our anti-blurred approach can improve the VO’s accuracy significantly when there are many blurred images. The whole closed-loop path of the dataset2 is also given in Figure 15a, which shows the walking distance of the dataset2 is around 300 m. The Figure 15b also presents the SIGD value of each frame in the path, which indicate images in that path have quite fluctuant degrees of the blurring.

In our second evaluation experiment for visual odometry with blurred images, we compare with Alberto Pretto’s algorithm [14], denote as AP in the following experiments, which improves the SIFT detector by recovering the PSF of the simple motion. In this experiment, we compare the AP with the SVO + A-B. In order to fair compare these two methods, the SVO used in this experiment will also employ SIFT as its detector, it is denoting as S_SVO. The experimental results on dataset1 and dataset2 are shown in Table 6 and Table 7 respectively.

The results in Table 6 and Table 7 show that the S_SVO + A-B can achieve much better performance than AP algorithm. As the AP algorithm needs to satisfy the assumption that the blurred motions of the images are linear, this may not be always satisfied in practice, especially when the robots walking on rough roads with non-linear illustration situations. Our method does not need the assumption of the linear blurred motion and can be applied in varied complex conditions containing blurred images.

In our third evaluation experiment for visual odometry with blurred images, we will evaluate the temporal complexity of our method. We calculate the average computation time per frame for each method in the dataset1 and dataset2, and the results are given in Table 8. The results in Table 8 show that our approach will not add too much computation time to the original VO algorithms, thus it can be well extended to the VO algorithms without too much additional computation cost.

## 5. Conclusions

This paper presents a visual odometry framework robust to the blurred images. Our approach can significantly improve the estimation accuracy of the visual odometry especially when there are severe blurred images in the robotic applications. As our approach does not try to deblur those blurred images and only tries to employ an anti-blurred key-frame selection, it can be widely applied to varied existed VO algorithms with a low additional temporal cost, which has been proved by the experiments.

In the future works, additional sensors such as the inertial sensors may be introduced into the anti-blurred VO framework to reduce the harm caused by the blurred images. And the illustration situations may also be included into our anti-blurred VO framework.

## Figures and Tables

**Table 1:** Estimated blurred degree on linear motion blurred images.

	Kernel Width	6	8	10	12	14
The image of *Bike*	SIGD	3.5660	3.9611	4.1767	4.4435	4.7866
	Marziliano	5.6197	6.4135	6.8119	7.3683	7.7798
	JNBM	3.8345	3.3307	3.2200	3.0336	2.9360
	CPBD	0.3723	0.3361	0.3182	0.2934	0.2690
The image of *Lighthouse*	SIGD	5.1517	5.3465	5.4536	5.5646	5.6864
	Marziliano	5.1273	5.4800	5.5926	5.6947	5.7927
	JNBM	4.6421	4.4427	4.1056	3.9374	3.6518
	CPBD	0.4227	0.3898	0.3893	0.3817	0.3592
The image of *Student sculpture*	SIGD	0.8578	1.1141	1.2545	1.4393	1.6730
	Marziliano	5.4835	5.8201	6.0052	6.2772	6.4567
	JNBM	2.7939	2.6311	2.4279	2.4126	2.3602
	CPBD	0.3627	0.3406	0.3336	0.3188	0.3137

**Table 2:** Estimated blurred degree on Gaussian blurred images.

	Standard Deviation	2	3	4	5	6
The image of *Bike*	SIGD	5.2605	5.8683	5.9879	6.0152	6.0260
	Marziliano	7.8843	9.4496	9.7682	9.7548	9.8992
	JNBM	2.6744	2.3291	2.1694	2.1420	2.1104
	CPBD	0.1030	0.0369	0.0424	0.0516	0.0616
The image of *Lighthouse*	SIGD	6.2327	6.7084	6.8292	6.8493	6.8612
	Marziliano	7.3760	7.9183	7.8618	7.7431	7.7386
	JNBM	3.0152	2.5630	2.5478	2.5360	2.4688
	CPBD	0.0468	0.0197	0.0272	0.0345	0.0431
The image of *Student sculpture*	SIGD	2.4278	3.4711	3.7582	3.8103	3.8267
	Marziliano	7.7243	8.7017	8.9335	8.7914	8.7945
	JNBM	1.9063	1.7128	1.6439	1.6008	1.5961
	CPBD	0.0511	0.01936	0.0349	0.0494	0.0661

**Table 3:** Estimated blurred degree on Gaussian blurred images.

	Rotated Angle	2	4	6	8	10
The image of *Bike*	SIGD	4.0737	4.8269	5.4614	5.9372	6.3040
	Marziliano	5.1828	5.9369	5.8818	5.7457	5.6422
	JNBM	3.7353	3.2516	2.8248	3.1235	3.4399
	CPBD	0.3969	0.3489	0.3459	0.3460	0.3517
The image of *Lighthouse*	SIGD	5.0180	5.4615	5.8601	6.2249	6.5511
	Marziliano	3.9805	4.1216	4.1470	4.2482	4.2178
	JNBM	6.3569	5.5806	5.2870	4.9411	5.2404
	CPBD	0.6158	0.5707	0.5565	0.5545	0.5584
The image of *Student sculpture*	SIGD	0.6326	1.1201	1.5630	1.9852	2.4048
	Marziliano	4.4000	4.6921	4.8826	5.0663	5.1374
	JNBM	3.4556	2.8975	2.5241	2.5212	2.4029
	CPBD	0.5386	0.5017	0.4871	0.4788	0.4748

**Table 4:** Experiment 1: The closed-loop error on dataset1.

VO Algorithm + Mode	Closed-Loop Error (m)
Libviso + F-B-F	3.0383
Libviso + K-F	2.9013
Libviso + A-B	1.7578
SVO + F-B-F	3.8892
SVO + K-F	3.5939
SVO + A-B	3.4804

**Table 5:** Experiment 1: The closed-loop error on dataset2.

VO Algorithm + Mode	Closed-Loop Error (m)
Libviso + F-B-F	21.2164
Libviso + K-F	14.0242
Libviso + A-B	6.5788
SVO + F-B-F	29.9466
SVO + K-F	14.3590
SVO + A-B	8.1435

**Table 6:** Experiment 2: The closed-loop error on dataset1.

VO Algorithm + Mode	Closed-Loop Error (m)
S_SVO + AP	6.1624
S_SVO + A-B	2.8586

**Table 7:** Experiment 2: The closed-loop error on dataset2.

VO Algorithm + Mode	Closed-Loop Error (m)
S_SVO + AP	28.8154
S_SVO + A-B	8.3554

**Table 8:** Experiment 3: Average Computation time.

Algorithm	Average Time (ms)
SVO	55
libviso	60
SVO + A-B	58.5
libviso + A-B	63.5

## Abbreviations

The following abbreviations are used in this manuscript:

VO: Visual Odometry
SIGD: Small Image Gradient Distribution
SLAM: Simultaneous Localization and Mapping
SFM: Structure From Motion
SBA: Sparse Bundle Adjustment
SIFT: Scale-invariant Feature Transform
PSF: Point Spread Function
JNB: Just Noticeable Blur
CPBD: Cumulative Probability of Blur Detection
